# Modulation of Wolframin Expression in Human Placenta during Pregnancy: Comparison among Physiological and Pathological States

**DOI:** 10.1155/2014/985478

**Published:** 2014-01-23

**Authors:** Angela Lucariello, Angelica Perna, Carmine Sellitto, Alfonso Baldi, Alessandro Iannaccone, Luigi Cobellis, Antonio De Luca, Maria De Falco

**Affiliations:** ^1^Department of Mental and Physical Health and Preventive Medicine, Section of Human Anatomy, Second University of Naples, Largo Madonna delle Grazie, 80138 Naples, Italy; ^2^Department of Sciences and Environmental, Biological and Pharmaceutical Technologies, Second University of Naples, Via Vivaldi 43, 81100 Caserta, Italy; ^3^Hamilton Eye Institute, University of Tennessee Health Science Center, 930 Madison Avenue, Memphis, TN 38163, USA; ^4^Department of Gynaecology, Obstetric and Reproductive Science, Second University of Naples, Via De Crecchio 4, 80138 Naples, Italy; ^5^Department of Biology, Section of Evolutionary and Comparative Biology, University of Naples “Federico II”, Via Mezzocannone 8, 80134 Naples, Italy; ^6^National Institute of Biostructures and Biosystems (INBB), Viale medaglie d'Oro 305, 00136 Rome, Italy

## Abstract

The *WFS1* gene, encoding a transmembrane glycoprotein of the endoplasmic reticulum called wolframin, is mutated in Wolfram syndrome, an autosomal recessive disorder defined by the association of diabetes mellitus, optic atrophy, and further organ abnormalities. Disruption of the *WFS1* gene in mice causes progressive **β**-cell loss in the pancreas and impaired stimulus-secretion coupling in insulin secretion. However, little is known about the physiological functions of this protein. We investigated the immunohistochemical expression of wolframin in human placenta throughout pregnancy in normal women and diabetic pregnant women. In normal placenta, there was a modulation of wolframin throughout pregnancy with a strong level of expression during the first trimester and a moderate level in the third trimester of gestation. In diabetic women, wolframin expression was strongly reduced in the third trimester of gestation. The pattern of expression of wolframin in normal placenta suggests that this protein may be required to sustain normal rates of cytotrophoblast cell proliferation during the first trimester of gestation. The decrease in wolframin expression in diabetic placenta suggests that this protein may participate in maintaining the physiologic glucose homeostasis in this organ.

## 1. Introduction

Wolfram syndrome, also known as DIDMOAD (diabetes insipidus, diabetes mellitus, optic atrophy, and deafness), is an autosomal recessive disorder [1] caused by more than 100 mutations in the *WFS1* gene, which was identified by positional cloning in 1998 [2–4]. Most are inactivating mutations, suggesting that loss of function may be responsible for the disease phenotype [5, 6]. Wolfram patients demonstrate noninflammatory atrophic changes in the brain [7] and in pancreatic islets, resulting in progressive diabetes, blindness, deafness, and other severe neurological defects [8, 9]. Consistent with these data, it has been demonstrated that the *WFS1* gene is expressed at very high levels in the brain and in pancreatic islets [10, 11].

In humans, wolframin expression was distributed in many organs, with different tissue and cell localization and expression levels [12]. In foetal systems, wolframin expression started faint and increased when development proceeded. In adult human tissues, a variable positive staining was observed in both simple and stratified epithelia [12].

Wolframin is a hydrophobic protein consisting of 890 amino acids with a molecular mass of ~100 kDa. This protein is a type II membrane protein with nine putative transmembrane segments [13] and large hydrophilic regions at both termini. Wolframin localizes primarily at the endoplasmic reticulum (ER) in a N_cyt_/C_lum_ membrane topology [3, 4, 10, 14, 15]. Specifically, wolframin colocalized with the ER marker protein disulphide isomerase (PDI) in unstimulated pancreatic *β*-cells [4]. Moreover, it has been demonstrated that glucose causes a wolframin translocation from the ER to the Golgi and stimulates the accumulation of wolframin on the plasma membrane, where it forms a complex with AC8-calmodulin and stimulates insulin biosynthesis and secretion [4].

Recently, it has been demonstrated that mice with a disrupted *WFS1* gene exhibited impaired glucose homeostasis and a selective *β*-cell loss [16]. In *WFS1*-deficient cells, glucose-stimulated elevation of the cytosolic Ca^2+^ concentration ([Ca^2+^]_cyt_) was impaired [6], and the ER-stress response was persistently activated [15, 17]. In addition, a recent report suggested that expression of wolframin in oocytes was associated with an increase in [Ca^2+^]_cyt_ and induced novel cation-selective channel activities in the ER membrane [9, 14]. These data suggested wolframin to play a role in cellular Ca^2+^ homeostasis and regulation of ER functions [15].

The ER, which constitutes the main intracellular Ca^2+^ storage area, plays a central role in Ca^2+^ homeostasis [15]. Various physiological and pathological conditions interfere with these functions, and overloading of these functions induces ER stress. Cells respond to such stress by activating several adaptive pathways, including induction of chaperones, attenuation of protein translation, and apoptosis, collectively called the unfolded protein response, or UPR [18]. Based on the ER localization of wolframin, it is reasonable to speculate that it may play a not yet defined role in the ER stress-induced cell death [14].

Apoptosis plays an important role in formation of human placenta [19–21]. Several investigators have demonstrated that the apoptotic rate increases progressively during normal gestation, which has been interpreted as part of normal placental development [22–25]. The formation of complexes between anti- and proapoptotic proteins appears to regulate cellular sensitivity to apoptosis [24, 26–29]. An abnormal level of apoptosis has been also correlated with a great variety of gestational pathologies such as miscarriages, ectopic pregnancy, intrauterine growth retardation, postterm pregnancy, preeclampsia, and diabetes [21–23, 30, 31].

Based on these premises, we set forth to investigate the expression of wolframin in human placenta throughout gestation in physiological conditions and contrasted it with the expression patterns in diabetes mellitus, a central characteristic of the Wolfram phenotype.

## 2. Materials and Methods

### 2.1. Samples

Human placental samples were obtained with informed consent from first trimester voluntary termination of gestation (VTG, *n* = 15), first trimester loss of pregnancy (LP, *n* = 15), following birth from a vaginal delivery (VD, *n* = 15), from caesarean sections (CS, *n* = 15), and from caesarean sections of patients with diabetes mellitus (DMP, *n* = 15). The gestation period ranged from 5 to 40 weeks. The specimens were immediately fixed in formalin for immunohistochemistry.

### 2.2. Immunohistochemistry

Immunohistochemistry was carried out essentially as described previously [32, 33]. Briefly, all sections were deparaffinized in xylene, rehydrated through a graded alcohol series, and washed in phosphate-buffered saline (PBS). PBS was used for all subsequent washes and for antiserum dilution. Tissue sections were quenched sequentially in 3% hydrogen peroxide and blocked with PBS-6% nonfat dry milk (Biorad) for 1 hr at room temperature. Slides then were incubated at 4°C overnight with an affinity-purified rabbit polyclonal immune serum raised against wolframin (803–240; developed by the Lesperance lab) at a 1 : 100 dilution and then with diluted anti-rabbit biotinylated antibody (Vector Laboratories) for 1 hr. All the slides then were processed by the ABC method (Vector Laboratories) for 30 min at room temperature. Novared (Vector Laboratories) was used as the final chromogen and hematoxylin was used as the nuclear counterstain. Negative controls for each tissue section were prepared by substituting the primary antiserum with the respective preimmune serum. All samples were processed under the same conditions. The expression level of wolframin-stained cells per field (250x) at light microscopy was calculated and compared in different specimens by two separate observers (A.B. and F.B.) in a double blind fashion and described as absent (○), very low (*◐*), low (●), moderate (●●), high (●●●), and very high (●●●●).

For each specimen, an HSCORE value was derived by summing the percentages of cells/areas stained at each intensity and multiplying that by the weighted intensity of the staining. An average of 22 fields was observed for each tissue by three observers at different times and the average score was used. All values were expressed as mean ± standard error of mean (SEM) and differences were compared by one-way analysis of variance (ANOVA) with SAS statistical software (SAS Institute, Cary, NC, USA). *P* values less than 0.05 were considered significant.

## 3. Results 

**Figure 1:**
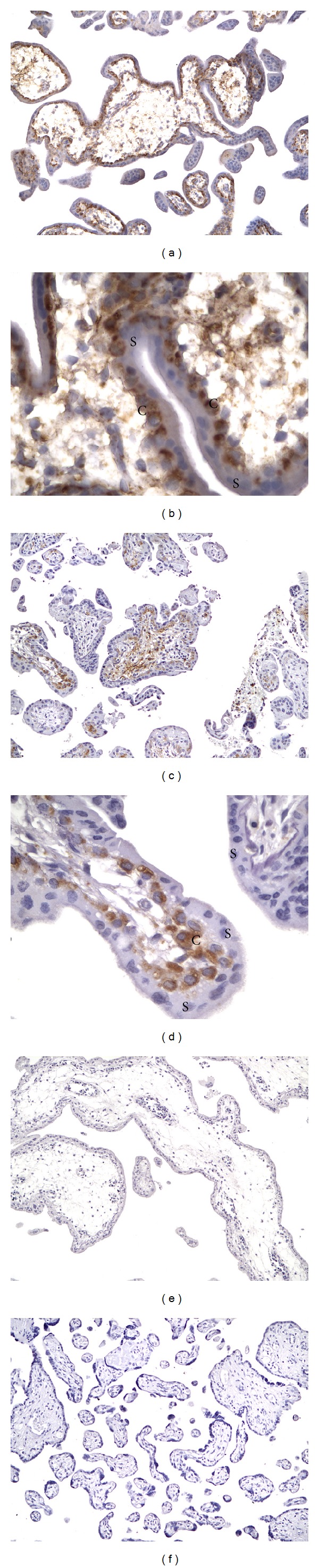
(a) Very high wolframin expression distributed in the cytotrophoblast cells of placental villi from the first trimester voluntary termination of gestation (VTG), 150x; (b) higher magnification showing the very high immunostaining for wolframin localized at perinuclear level in the cytotrophoblast cells from VTG, 640x; (c) moderate wolframin expression in the cytotrophoblast cells and in the stroma of placental villi from loss of pregnancy (LP), 150x; (d) higher magnification showing the moderate wolframin immunopositivity in the cytoplasm of cytotrophoblast cells from LP, 640x; (e) representative negative control of the first trimester VTG, 150x; (f) representative negative control of the third trimester vaginal delivery (VD), 150x. For all the images, C represents cytotrophoblast and S represents syncytiotrophoblast.

**Figure 2:**
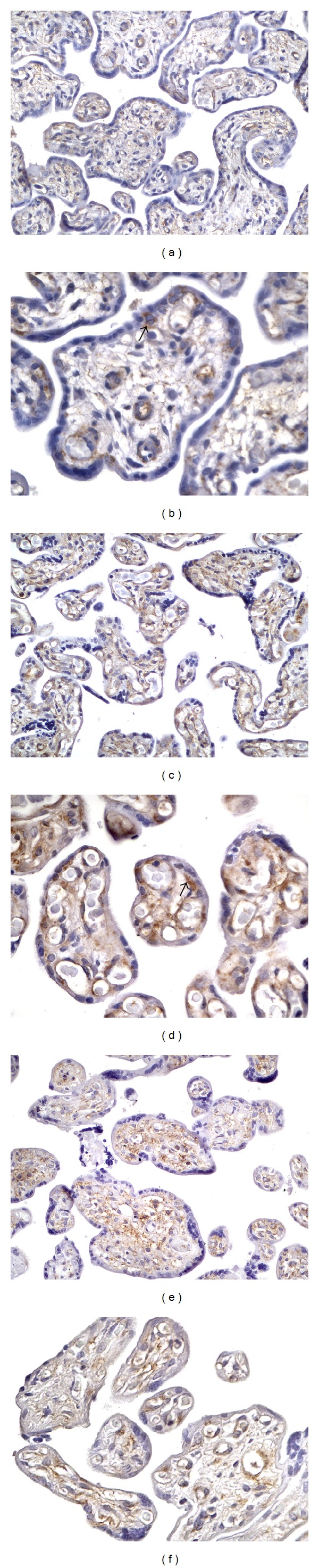
(a) Low wolframin expression in the endothelial cells inside placental villi from the third trimester vaginal delivery (VD), 300x; (b) higher magnification showing wolframin immunopositivity localized in the cytotrophoblast cells (arrow) from the VD, 640x; (c) moderate wolframin immunopositivity in the endothelial cells lining blood vessels of placental villi from caesarean section (CS), 300x; (d) higher magnification showing moderate wolframin expression in endothelial cells and in cytotrophoblast cells (arrow) of placental villi from CS, 640x; (e) low expression level of wolframin in the stroma and endothelial cells of the third trimester placental villi from diabetic women, 300x; (f) higher magnification showing low wolframin expression localized almost exclusively in the stroma and endothelial cells of placenta from diabetic women; no immunopositivity was shown in both cytotrophoblast and syncytiotrophoblast.

By immunohistochemical criteria, we observed a modulation of human placental wolframin expression from the first to the third trimester of gestation. Specifically, we showed a high wolframin expression in cytotrophoblast cells, the inner proliferative layer during the first trimester of gestation (Figure 1). Wolframin expression decreased to a moderate level of expression in the third trimester of gestation, at which time it mainly localized in the stroma and in the endothelial cells of placental villi (Figure 2). In diabetic women, the decrease in wolframin placental expression observed in the third trimester of gestation was greater than in healthy women, diminishing from a moderate to a low expression level (Figure 2).

In addition, we characterized the modulation of wolframin expression in human placenta throughout the gestation (Tables 1 and 2). In the first trimester VTG placentas, we observed a very high wolframin expression in the cytoplasm of the cytotrophoblast cells forming the inner layer of placental villi (Figure 1(a)). In these cells, wolframin was localized at the perinuclear level (Figure 1(b)). Unlike these cells, the syncytiotrophoblast, the outer differentiated layer of placental villi, appeared completely negative for this protein. In the first trimester LP placentas, wolframin expression slightly decreased to a moderate/high level of expression in the cytotrophoblast cells, whereas a moderate/high immunopositivity for this protein was observed in the stroma of placental villi (Figures 1(c) and 1(d)). In VD placentas, we observed a low wolframin expression distributed in the stroma and the endothelial cells lining blood vessels inside placental villi (Figure 2(a)). Low wolframin immunopositivity was also detectable in the cytoplasm of some cytotrophoblast cells (Figure 2(b)). Syncytiotrophoblast appeared to be wolframin-negative. In CS placentas, we observed an increased wolframin expression to a moderate level of expression, with localization mainly at the endothelial cells and to some cytotrophoblast cells of placental villi (Figures 2(c) and 2(d)). Also in this case, syncytiotrophoblast appeared negative for this protein. On the contrary, in placentas of the third trimester of gestation from DMP women, we observed a decreased wolframin expression to a low expression level in the stroma and in the endothelial cells (Figures 2(e) and 2(f)). Both cytotrophoblast and syncytiotrophoblast cells appeared completely negative for this protein.

**Figure 3:**
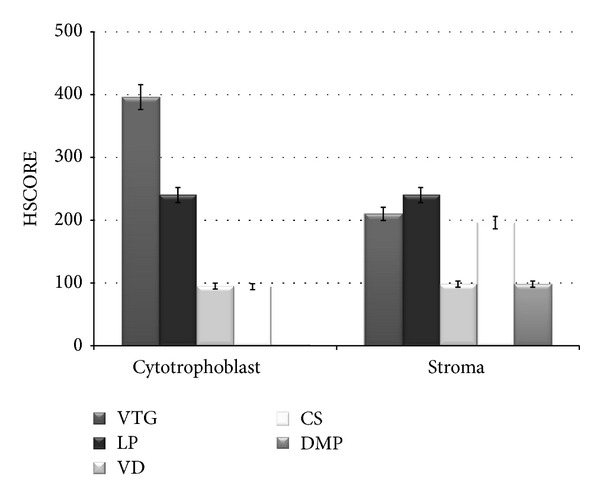
Intensity staining of wolframin immunopositivity in human placenta during gestation. Wolframin expression level in placental villi from physiological and pathological human placentas during gestation is shown. Vertical lines show S.E.M. VTG: voluntary termination of gestation, LP: loss of pregnancy, VD: vaginal delivery, CS: caesarean section, and DM: third trimester placentas from women with diabetes mellitus.

Table 2 and Figure 3 illustrate a comparison in the expression patterns of wolframin immunopositivity in normal human placenta versus diabetic gestation, as ascertained by HSCORE and immunohistochemical staining intensity analysis. We observed a very high expression level of wolframin in the cytotrophoblast cells of placentae of the first trimester in first trimester VTG specimens, compared to a low/moderate expression level of wolframin in the cytotrophoblast cells of the third trimester placentae of both VD and CS births. Interestingly, in diabetic women, we did not observe any wolframin expression in the cytotrophoblast cells of the third trimester placental villi. In addition, wolframin expression in the stroma and endothelial cells did not show any significant differences between the first and the third trimester of gestation in both physiological conditions and DMP.

## 4. Discussion

Wolframin acts by regulating cellular Ca^2+^ homeostasis, at least in part by modulating ER Ca^2+^ concentrations [15]. Defective ER Ca^2+^ homeostasis, mutations in ER resident proteins, and/or abnormalities of the ER-associated degradation (ERAD) system activate the UPR, in which cells respond by inducing chaperones, attenuating protein translation, and inducing apoptosis [14]. The increase in wolframin expression is attributable, at least in part, to enhanced *WFS1* promoter activity stimulated by ER stress-inducing chemicals, thus indicating a direct link between wolframin function and ER stress responses [14]. Therefore, it has been suggested that wolframin plays its physiological role in protecting cells from ER stress-induced apoptosis [14]. Consistent with this hypothesis, it has been demonstrated that *WFS1 *deficiency increases caspase-3 cleavage and decreases *β*-cell proliferation [17].

Recently, several studies have suggested that apoptosis plays an important role in the normal development, remodeling, and aging of placenta [34]. In addition, it has been demonstrated that apoptosis increases in pregnancies complicated by some disorders such as preeclampsia, foetal growth restriction, and diabetes [21, 30, 31, 35, 36]. The placenta mediates the nutrition to the growing foetus, provides a barrier from maternal pathogens, and protects the foetus from maternal rejection [37, 38]. To this end, two different cell populations characterize the villous trophoblast: an inner proliferative layer, the cytotrophoblast, that displays highly proliferative and invasive properties and an outer differentiated layer, the syncytiotrophoblast, displaying various functions necessary to the maintenance of pregnancy and foetal growth with little potential for proliferation [39].

Many molecules are associated with the induction and prevention of apoptosis in different models [21, 31, 40, 41]. Kim et al. [42] and De Falco et al. [19, 24] have demonstrated that the expression of the antiapoptotic factor, Bcl-2, diminishes as gestation progresses, suggesting that a parturition-associated biological change might induce apoptosis in the placental villi [31]. Recently, we have compared the expression of the proapoptotic Bax protein between first trimester VTGs and first trimester LPs, showing a strong increase of Bax expression in the latter compared to the low/moderate Bax immunopositivity in the former, thereby suggesting that upregulation of apoptosis may be involved in pregnancy complications leading to miscarriage. In addition, we have observed a more intense Bax expression in third trimester VD compared to third trimester CS, which suggests that apoptosis in placental villi may be a key factor in allowing normal delivery [21].

In the present paper, we have first demonstrated that wolframin expression decreases as pregnancy progresses, in accordance with the expression trend of other antiapoptotic factors like Bcl-2 [24]. In addition, we have observed a further decrease in wolframin expression in third trimester placentae from diabetic women, suggesting an important role of this protein in glucose homeostasis also in human placenta. Furthermore, we have observed that wolframin expression decreased in LP during the first trimester compared to the intense wolframin expression observed in the first trimester voluntary termination of gestation. This trend of wolframin expression in human placenta was opposite to that of Bax protein [21]. With respect to the third trimester of gestation, we observed an increase of wolframin expression in CS specimens compared to the VD ones, which is again opposite to the pattern of Bax expression [21]. Moreover, we have observed that, in DMP of the third trimester of gestation, wolframin expression strongly decreases in all the placental compartments. Wolframin was reported to negatively regulate activating transcription factor 6*α*, a key transcription factor implicated in ER stress signaling, through the ubiquitin-proteasome pathway [16, 43, 44]. This supposed cellular function indicates its important role in cell homeostasis, so decrease of wolframin expression in DMP shows that deregulation processes take place in diabetic women.

## 5. Conclusions

Our results suggest an important role of wolframin in controlling apoptotic events in cytotrophoblast cells of human placenta throughout gestation. These observations can be considered an interesting basis to further investigate subcellular pathways that human placental cells activate in response to physiological and pathological stimuli during gestation.

## Figures and Tables

**Table 1:** Wolframin expression in human placenta during gestation.

	Cytotrophoblast	Syncytiotrophoblast	Endothelial cells	Stroma
Voluntary termination of gestation (VTG)	●●●●	○	●●●	●●●
Loss of pregnancy (LP)	●●*◐*	○	●●	●●*◐*
Vaginal delivery (VD)	●	○	●	●
Caesarean sections (CS)	●●	○	●●	●●
Diabetes mellitus complicated patients (DMP)	○	○	●	●

○: absent; *◐*: very low; ●: low; ●●: moderate; ●●●: high; ●●●●: very high.

**Table 2:** Wolframin HSCORE values in human placenta during gestation.

	VTG	LP	VD	CS	DMP
Cytotrophoblast	396 ± 19	212 ± 14	95 ± 6	94 ± 5	0
Stroma	210 ± 10	240 ± 12	98 ± 7	196 ± 10	98 ± 9

All values were expressed as mean ± standard error of mean (SEM).
